# Calculation of Kidney Volumes with Magnetic Resonance in Patients with Autosomal Dominant Polycystic Kidney Disease: Comparison between Methods

**DOI:** 10.3390/diagnostics13233573

**Published:** 2023-11-30

**Authors:** Stefano Di Pietro, Alfredo Gaetano Torcitto, Carmelita Marcantoni, Gabriele Giordano, Christian Campisi, Giovanni Failla, Licia Saporito, Rosa Giunta, Massimiliano Veroux, Pietro Valerio Foti, Stefano Palmucci, Antonio Basile

**Affiliations:** 1 Radiology Unit 1, Department of Medical Surgical Sciences and Advanced Technologies “GF Ingrassia”, University Hospital Policlinico “G. Rodolico-San Marco”, University of Catania, 95123 Catania, Italy; s.dipietro@studium.unict.it (S.D.P.); a.torcitto@gmail.com (A.G.T.); gabriele.21.giordano.01@gmail.com (G.G.); christiancampisi22@gmail.com (C.C.); failla.giovanni@gmail.com (G.F.); pietrofoti@hotmail.com (P.V.F.); basile.antonello73@gmail.com (A.B.); 2 UOSD of Nephrology and Dialysis, University Hospital Policlinico “G. Rodolico-San Marco”, 95123 Catania, Italy; marcantoni.carmelita@gmail.com (C.M.); licia.saporito@email.it (L.S.); rosa_giunta@virgilio.it (R.G.); 3 Organ Transplant Unit, Department of Medical Surgical Sciences and Advanced Technologies “GF Ingrassia”, University Hospital Policlinico “G. Rodolico-San Marco”, University of Catania, 95123 Catania, Italy; veroux@unict.it; 4 UOSD “IPTRA”, Department of Medical Surgical Sciences and Advanced Technologies “GF Ingrassia”, University Hospital Policlinico “G. Rodolico-San Marco”, University of Catania, 95123 Catania, Italy

**Keywords:** kidney, magnetic resonance, ADPKD

## Abstract

Autosomal dominant polycystic renal disease (ADPKD) is the most frequent kidney inheritable disease, characterized by the presence of numerous bilateral renal cysts, causing a progressive increase in total kidney volume (TKV) and a progressive loss of renal function. Several methods can be used to measure TKV by using MRI, and they differ in complexity, accuracy and time consumption. This study was performed to assess the performance of the ellipsoid method and the semi-automatic segmentation method, both for TKV and SKV (single kidney volume) computation. In total, 40 patients were enrolled, and 78 polycystic kidneys analyzed. Two independent operators with different levels of experience evaluated renal volumetry using both methods. Mean error for ellipsoid method for SKV computation was −2.74 ± 11.79% and 3.25 ± 10.02% for the expert and the beginner operator, respectively (*p* = 0.0008). A Wilcoxon test showed a statistically significant difference between the two operators for both methods (SKV *p* = 0.0371 and 0.0034; TKV *p* = 0.0416 and 0.0171 for the expert and the beginner operator, respectively). No inter-operator significant difference was found for the semi-automatic method, in contrast to the ellipsoid method. Both with a Wilcoxon test and Bland–Altman plot, statistically significant differences were found when comparing SKV and TKV measurements obtained with the two methods for both operators, even if the differences are stronger for the beginner operator than for the expert one. The semi-automatic segmentation method showed more inter-observer reproducibility. The ellipsoid method, in contrast, appears to be affected by greater inter-observer variability, especially when performed by operators with limited experience.

## 1. Introduction

Autosomal dominant polycystic renal disease (ADPKD) is the most frequent inheritable kidney disease [1,2,3]. The disease is characterized by the presence of numerous bilateral renal cysts, whose development begins in utero and slowly continues over time, causing a progressive increase in kidney volume and a progressive loss of renal function, which becomes clinically manifest, in most cases, between the fourth and the sixth decade of life [4,5].

The disease causes a progressive decline in renal function, which results in a reduction in glomerular filtration rate (GFR). ADPKD, therefore, is the fourth cause of end-stage renal disease (ESRD), representing about 10–15% of patients on dialysis [6] and 10% of cases requiring renal replacement therapy [1,2,3].

Mutations in the PKD1 gene, localized on the short arm of chromosome 16, are responsible for 80% of ADPKD cases, while mutations in the PKD2 gene, localized on the long arm of chromosome 4, are attributed to 15% of cases. The remaining 5% of cases are due to rare mutations in other loci, which are yet unknown [5]. Mutations in PKD1 or PKD2 lead to the lack of function of the proteins encoded by these genes, which are essential for maintaining the normal structure of the renal tubular architecture. These cellular functions will be altered with consequent development of renal cysts. Cysts will progressively increase in number and size throughout the patient’s life and, at the same time, there will be a progressive subversion of the renal parenchyma with an increase in the total kidney volume (TKV). This structural subversion is associated with a gradual loss of renal function, up to the ESRD. ESRD occurs in approximately 50% of patients, generally occurring around the fifth decade of life, with significant inter-individual variability [7,8]. The typical clinical presentation includes signs and symptoms such as early onset hypertension, abdominal pain, haematuria, urinary tract infections and nephrolithiasis. This kind of presentation can usually be observed several years before the onset of renal insufficiency, sometimes even in childhood [5].

Imaging plays a central role both in the diagnosis and the monitoring of the disease, and in the evaluation of treatment response. It is also useful in identifying and managing complications in ADPKD patients. Magnetic resonance imaging today is the reference technique in ADPKD. It is the gold standard for radiological diagnosis, for follow-up, and for the assessment of ADPKD and its complications [9].

The importance of the role played by MRI emerges mainly in the assessment of ADPKD progression, since it represents the method of choice for that purpose. The CRISP (Consortium of Radiologic Imaging Studies of PKD) study, in fact, has shown that MRI is the best technique to study renal volumetry and that the measurement of total kidney volume is today the gold standard for assessing ADPKD progression, for estimating the risk of end-stage renal disease, and for monitoring the effectiveness of pharmacological treatment in slowing the disease’s progression [10,11]. Several studies [11,12,13,14,15,16,17], in fact, have shown that the most important prognostic biomarker to assess the progression of ADPKD and to predict the risk of ESRD is the total kidney volume, and the Mayo imaging classification [18] is currently considered as the best model used to predict the time of onset of ESRD, stratifying patients affected by ADPKD into different classes (from 1A through 1E) according to height-adjusted total kidney volume for age.

Several methods can be used to measure TKV using MRI, and they differ in complexity, accuracy, precision and time consumption [19].

Based on current knowledge, which emphasizes the importance of the kidney volume in Autosomal Dominant Polycystic Kidney Disease, and recognizes MRI as best imaging tool to assess renal measurements, the aim of our study is to compare the ellipsoid method to the semi-automatic volumetric segmentation method for SKV (single kidney volume) and TKV (total kidney volume) computation. This work was performed to assess the performance, evaluated in terms of accuracy and mean error, of the ellipsoid method compared to the semi-automatic volumetric segmentation method, which was the reference method; this is because it is more similar to the manual method [19], and it allows us to analyse the inter-observer agreement for both methods and both SKV and TKV values.

Furthermore, we compared the results obtained in our experience with those obtained in similar studies in the literature.

## 2. Materials and Methods

### 2.1. Population Study

This study was performed from March 2016 to June 2022. A total of 40 patients were enrolled; 7 were treated with kidney transplant. The size of native kidneys was analysed for a total number of 78 kidneys; 2 patients had a solitary native kidney after transplantation. The study population included 18 males (45%) and 22 females (55%), ranging between 27 and 78 years of age (mean age of 48.8 ± 10.5).

The characteristics of the study sample are summarized in Table 1.

### 2.2. MR Protocol

All MRI examinations were performed using a 1.5 Tesla MR scanner (Signa HDx MR System; GE Healthcare, Milwaukee, WI, USA), and they were acquired using a high resolution 8-channel phased array coil. Sequences were respiratory-triggered, so a “respiratory” belt was used. MR imaging was performed with patient in the supine position (feet first). Patients observed no specific diet restrictions or hydration prior to MR examinations. No intravenous hypotonic agent was administrated. All patients had provided written consensus prior to the MR examination. Acquisition plans included the entire renal volume, and all sequences were acquired in the expiratory phase.

Our study protocol is summarized in Table 2.

### 2.3. Image Analysis

All kidney volume computations were performed independently by two operators with different levels of experience, using both ellipsoid and semi-automatic volumetric segmentation methods. The first operator had more experience with kidney volume computation, while the second operator was a beginner, as he had started to perform kidney volume computation for the purposes of this study after specific training.

**Figure 1:**
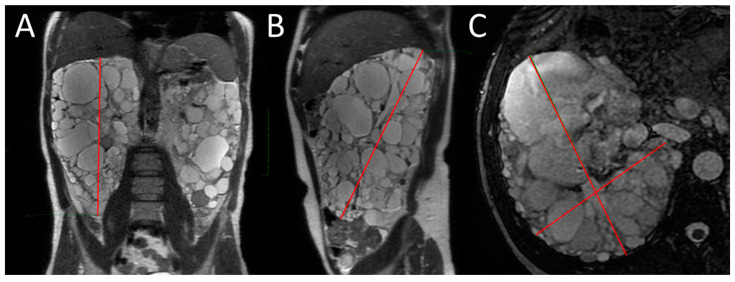
MRI measurement of renal diameters on the right kidney of a patient with ADPKD for SKV computation using the ellipsoid method. Coronal length (**A**), sagittal length (**B**), width and depth (**C**).

**Figure 2:**
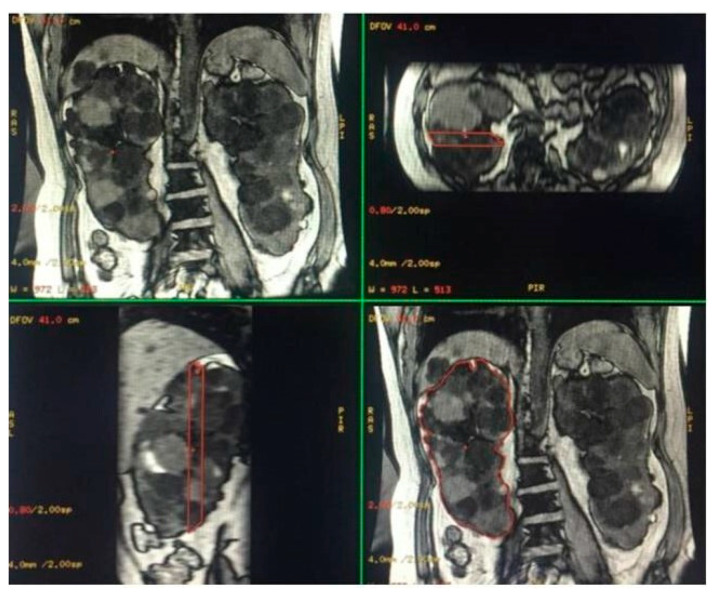
Semi-automatic volumetric segmentation. View of images in the three planes of space during segmentation.

**Figure 3:**
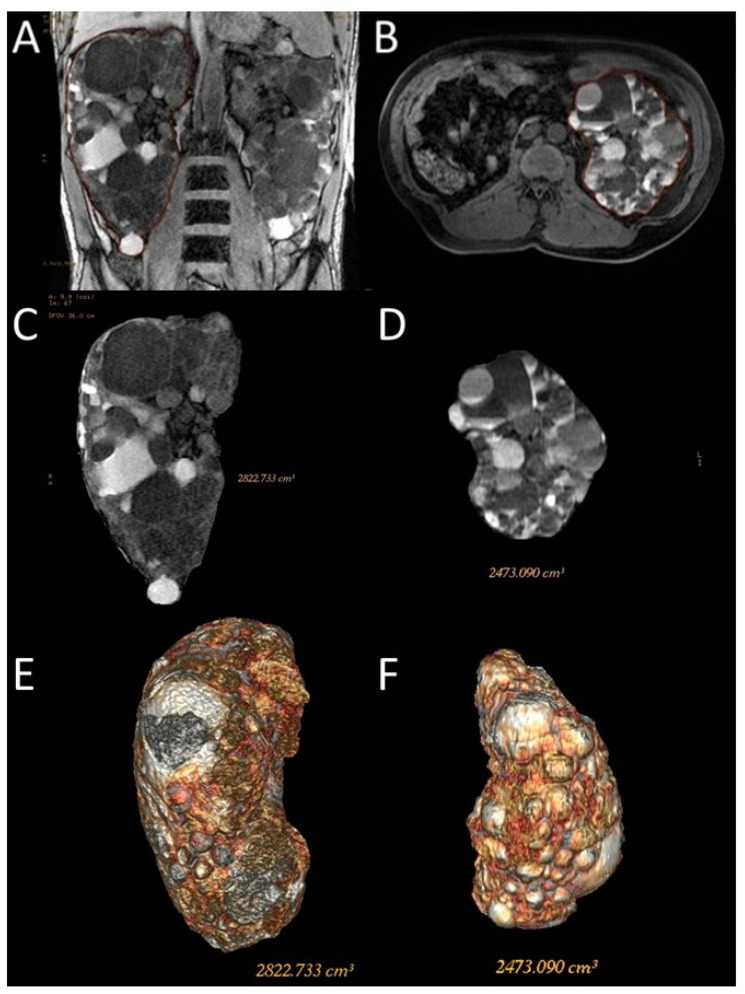
Representative images of the semi-automatic volumetric segmentation method performed on two different patients. This method requires manual tracing of renal contours on some images—Coronal 3D Dual Echo (**A**) or axial LAVA (**B**)—with automatic reconstruction in the intermediate images. Extraction of the segmented kidney volume with SKV computation on the same contour plane (**C**,**D**) and reconstruction in volume rendering (**E**,**F**).

SSFP and SSFSE acquisition planes were used for the evaluation of kidney volume through the ellipsoid method (Figure 1). The diameters of kidneys were measured in millimetres, and the online calculator tool developed by the Mayo Clinic and freely available on the Mayo website [14,18] was used to calculate the SKV and the TKV. For patients with only one native kidney, TKV coincided with the volume of the single kidney. Volumetric segmentation (Figure 2 and Figure 3) was performed using a dedicated workstation (Advantage Workstation 4.6 General Electric; GE Healthcare, Milwaukee, WI, USA).

For this type of segmentation, the 3D sequences were selected; Coronal 3D T1 Dual Echo acquisitions were preferred for the shorter time requirement for semi-automatic segmentation; however, sometimes, due to the presence of motion artifacts or for greater operator confidence, the axial LAVA sequences were used. Both operators used the same sequence on the same patient to perform the semi-automatic volumetric segmentation.

### 2.4. Statistical Analysis

Statistical analysis was performed using the MedCalc program (MedCalc version 18.2.1, MedCalc Software bvba©, Mariakerke, Belgium). Normal distribution of data was assessed using Q–Q plots and a Shapiro–Wilk test.

TKV and SKV values obtained via the ellipsoid method were referred to as eTKV and eSKV, respectively, while 3dTKV and 3dSKV were used to indicate TKV and SKV values obtained using the semi-automatic volumetric segmentation method.

The performance of the ellipsoid method compared to the semi-automatic volumetric segmentation method was assessed using the mean error, which was calculated separately for both the observers and for the average of the values obtained by the two observers, and it is expressed as the mean percentage difference.

The following formulas were used:

(3dSKV − eSKV)/3dSKV × 100%

(3dTKV − eTKV)/3dTKV × 100%

Positive values indicate that the ellipsoid method underestimates the kidney volume. The resulting values were then compared using Student’s *t*-test.

Comparisons between the two measurement methods (ellipsoid and semi-automatic volumetric segmentation) and between the values obtained by the two operators were performed using the Wilcoxon test, both for SKV and TKV. In addition, the Bland–Altman plot was used for method comparison.

## 3. Results

### 3.1. Performance of the Ellipsoid Method

The mean percentage error for eSKV and eTKV showed significative differences between the two operators. Data are summarized in Table 3.

The mean percentage errors for the ellipsoid method for SKV computation were −2.74 ± 11.79% and 3.25 ± 10.02% for the expert and the beginner operator, respectively, with a *p*-value of 0.0008 calculated using Student’s *t*-test. For TKV computation, the data show that mean percentage errors were −2.6 ± 8.74% and 3.41 ± 8.29% for the expert and the beginner operator, respectively, with a *p*-value of 0.0023 calculated using Student’s *t*-test. The mean percentage errors derived from the average of the values obtained by the ellipsoid method were almost comparable (0.27 ± 9.79% for SKV and 0.41 ± 7.76% for TKV computation, respectively).

### 3.2. Wilcoxon Test for Comparisons between Methods and Operators

Since the non-normality of data has been shown by drawing Q-Q Plots and by performing the Shapiro–Wilk test, values are given as median with interquartile range (IQR), and measurements were analyzed using the Wilcoxon test for paired samples to check for statistically significant differences between the kidney volume measurements obtained using the ellipsoid and the semi-automatic volumetric segmentation method, and between the measurements obtained by both operators.

The median SKV and IQRs (25–75%) obtained via the semi-automatic volumetric segmentation method were 714.61 mL (487.18–1385.84) and 696.03 mL (503.01–1419.82) for Operator 1 and Operator 2, respectively. The median TKV and IQRs (25–75%) obtained via the semi-automatic volumetric segmentation method were 1365.92 mL (1010.97–2667.1) and 1355.3 mL (1052.24–2812.94) for Operator 1 and Operator 2, respectively. The median SKV and IQRs (25–75%) obtained via the ellipsoid method were 715.75 mL (500.4–1421.6) and 713.9 mL (468.3–1313.7) for Operator 1 and Operator 2, respectively. The median TKV and IQRs (25–75%) obtained via the ellipsoid method were 1388.05 mL (1006.5–2677) and 1314.15 mL (974.7–2679.5) for Operator 1 and Operator 2, respectively.

The results of the Wilcoxon analysis are shown in Table 4 and Table 5. A *p*-value < 0.05 indicates that there is a statistically significant difference.

The Wilcoxon analysis showed that there were statistically significant differences between the kidney volume values measured by Operator 1 in performing the ellipsoid method and the semi-automatic volumetric segmentation method (*p*-values of 0.0371 and 0.0416 for SKV and TKV, respectively); there were also statistically significant differences between kidney volume values obtained via the two methods by Operator 2 for SKV and TKV (*p*-values of 0.0034 and 0.0171 for SKV and TKV, respectively).

The Wilcoxon test for paired samples performed between the kidney volume values obtained by both operators using each method showed statistically significant differences between the values obtained via the ellipsoid method from each operator (*p*-values < 0.0001 for both SKV and TKV), while there were no significant differences between the values obtained via the semi-automatic volumetric segmentation method (*p*-values of 0.7742 and 0.948 for SKV and TKV, respectively).

### 3.3. Comparison between Methods Using Bland–Altman Plots

**Figure 4:**
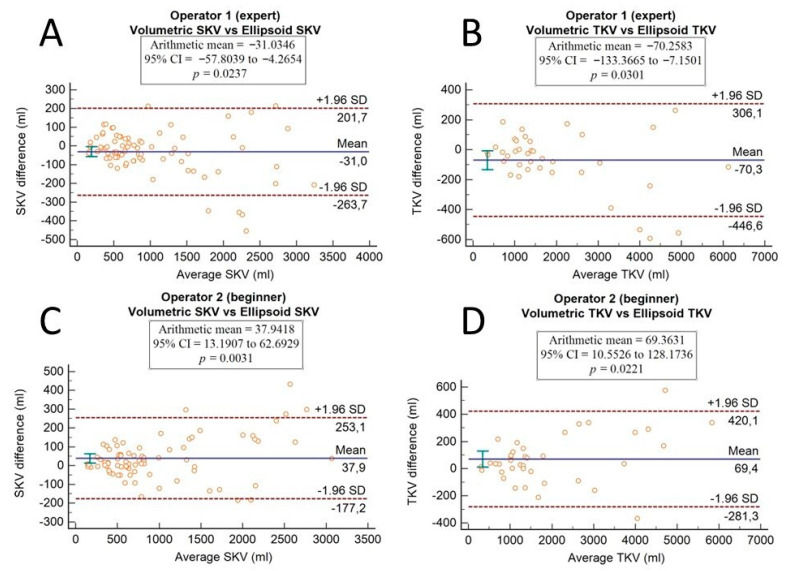
Bland–Altman plots show the agreement between the ellipsoid method and the semi-automatic volumetric segmentation method. Differences in single kidney volume and in total kidney volume are plotted against average SKV and TKV values of the two methods for the expert operator (**A**,**B**) and for the beginner operator (**C**,**D**). A *p*-value < 0.05 indicates that there is a significant systematic difference.

The agreement between the ellipsoid method and the semi-automatic volumetric segmentation method is shown in Bland–Altman plots, which were drawn separately for both operators (Figure 4).

## 4. Discussion

This study compared the ellipsoid method to the semi-automatic volumetric segmentation method for SKV and TKV computation, in terms of accuracy and mean error. Furthermore, we evaluated the interobserver variability for both methods.

The experimental set included 40 patients for a total number of 78 kidneys (since 2 patients had a solitary native kidney), with a very large range of single kidney volume (from 138 to 3139 mL). Two independent operators with different levels of experience measured the volume of each kidney using both methods.

As many authors have shown (Sharma et al. [20], Turco et al. [21], Magistroni et al. [19], Zöllner et al. [22], Christensen et al. [23], Seuss et al. [24]), and even in our experience, manual or semi-automatic volumetric segmentation methods tend to be much more time-consuming than the ellipsoid method. The ellipsoid method is the simplest method of estimating TKV, since it only requires the measurement of sagittal and coronal lengths, (the width and depth of the kidneys), and then the total volume of each kidney is calculated using the ellipsoid formula.

The manual segmentation method differs in complexity, because based on 2D MR acquisitions, it requires the manual tracing of the kidney’s contour in each slice, and the kidney volume is then calculated by multiplying the area of the kidney in each slice by the slice thickness and summing all the partial volumes [25].

In this study, we used the semi-automatic volumetric segmentation approach, which requires the manual tracing of kidney contour in some slices of 3D MR acquisitions with automatic reconstruction in the intermediate images. Dedicated software then extracts the kidney volume.

We have assumed that the semi-automatic volumetric segmentation method is comparable to the manual segmentation method because, in the case of a software error in the reconstruction of the intermediate contours, the operators have always carried out a manual correction of the errors, thus causing the two methods almost to overlap. For this reason, in our work, the semi-automatic volumetric segmentation method was considered the reference method.

Furthermore, the more complex execution technique can intuitively explain why manual and semi-automatic volumetric segmentation methods require much more time than the ellipsoid one. This method, moreover, requires the use of dedicated equipment (i.e., software or workstations). Thus, this method being so laborious, its use in clinical care is limited. Despite these limitations, the manual segmentation method is the reference standard for TKV computation [26].

Due to the need to find more straightforward, less laborious, but reasonably reliable methods, researchers have been and still are interested in testing TKV estimation methods that include the ellipsoid method and comparing them to the manual segmentation method (sometimes named the “planimetry” method [19,20]).

Cheong et al. [27], in an MRI-based study, used ex vivo and in vivo models and a retrospective group of 150 clinically referred patients to test the validity of the ellipsoid formula for estimating kidney volume, comparing it to the manual segmentation method, which was denoted the “disc-summation method”. Furthermore, they used the water displacement method to obtain an independent determination of the kidney volume. Their results showed that the renal volume obtained via the manual segmentation method was within 5% of the volume that was determined using the water displacement method, which was considered the “true kidney volume”, corroborating that the manual segmentation method is highly reliable for kidney volume computations, while the ellipsoid method consistently and significantly underestimate the correct kidney volume (*p* < 0.0001), with a mean percentage error of eSKV versus mSKV of 18% in men and 15% in women.

Higashihara et al. [28], moreover, agree with previous reports regarding the ellipsoid method tends to underestimate kidney volume, compared to volumetric measurements.

Mean percentage errors of eSKV and eTKV values calculated separately for the two operators were quite different, since when the beginner operator performed the ellipsoid method, the mean percentage error was 3.25% ± 10.02% for SKV and 3.41% ± 8.29% for TKV; meanwhile, when the expert operator performed it, the mean percentage error was −2.74% ± 11.79% for SKV and −2.6% ± 8.74% for TKV. This demonstrates the influence of experience in the use of the ellipsoid method. The beginner operator tended to underestimate the kidney volume with this method, reaching values comparable to those reported in the literature by Cheong et al. [27]. The expert operator, on the other hand, obtained more comparable measurements with both methods.

Moreover, the Wilcoxon test for paired samples showed that there were no statistically significant differences between the SKV and TKV values obtained via the semi-automatic volumetric segmentation method by both operators (*p*-values of 0.7742 and 0.948 for SKV and TKV, respectively), indicating that the kidney volume measurements obtained using this method are highly reproducible. For the ellipsoid method, in contrast, the Wilcoxon analysis showed that there were statistically significant differences between the two operators, both for SKV and TKV (*p*-values < 0.0001), indicating that this method has less reliability.

Our experience, therefore, showed that the ellipsoid method is more operator-dependent than the semi-automatic volumetric segmentation method.

In agreement with our results, Spithoven et al. [29], in a cross-sectional and longitudinal diagnostic test study, compared the mid-slice method and the ellipsoid method to the manual tracing method, which was the gold-standard method. They found that the variability in eTKV obtained via ellipsoid method was significantly higher compared to manual TKV, since the average intraobserver and interobserver coefficient variations were 1.8% and 2.3% for TKV measured using the manual tracing method, respectively. The coefficient variations for TKV estimated via the ellipsoid method, instead, were significantly higher than for the manual tracing method (3.9% and 6.3%, respectively). However, our results can be considered consistent with values reported by Spithoven et al. [29].

The results of this study are also in line with those of Sharma et al., who conducted a recently published study [20] with the purpose of identifying the most efficient kidney volume computation method to be used in clinical studies evaluating the effectiveness of treatments on ADPKD progression. For Sharma et al. [20], the manual segmentation method showed higher reproducibility, more accuracy and more precision than the ellipsoid method, which had higher intra-and interobserver variability. The study adopted the “ImageJ polyline” (which is essentially a manual segmentation approach), as the reference method, and various MRI- and CT-based methods were compared to it, including the ellipsoid method and “Osirix free hand” (another manual segmentation approach, essentially), which showed the highest agreement with “ImageJ polyline”, and high accuracy (mean difference of −0.8%). Simplified methods (which included the ellipsoid method), by contrast, showed the lowest accuracy, precision, and difference in SKV between the ellipsoid method and “ImageJ polyline” (mean of −18.8%), with *p* < 0.01. Furthermore, planimetry methods showed the highest interobserver agreement, while the ellipsoid method had the lowest reproducibility.

The comparison between methods (performed with the Wilcoxon test for paired samples) and the Bland–Altman plots showed that there were statistically significant differences between both operators and both SKV (*p*-values of 0.0371 and 0.0034) and TKV (*p*-values of 0.0416 and 0.0171) computation.

Coincident results were obtained via a comparison between methods performed with Bland–Altman plots. For the expert operator, there were significant systematic differences in both SKV and TKV computation (*p*-values of 0.0237 and 0.0301, respectively); meanwhile, for the beginner operator, *p*-values were of 0.0031 and 0.0221 for SKV and TKV, respectively. Based on these data, there was a significant systematic difference between methods for both operators, even if the differences are stronger for the beginner operator than for the expert one, who seemed to be able to obtain more reliable measurements with the ellipsoidal method.

Our results, therefore, could suggest that the performance of the ellipsoid method strongly depends on the experience of the operator, and that it is a useful method for estimating TKV with acceptable reliability only when performed by an operator with adequate experience.

The performance of the ellipsoid method was evaluated even by Turco et al. [21], who performed a study in which several methods for TKV computation in ADPKD patients were tested, evaluated, and compared to the reference manual segmentation method applied to MR images. In this study, the Intraclass Correlation Coefficients for TKV from MRI using the ellipsoid method were 0.977 for both the right and left kidney, while variability analysis, in line with our results, showed that the ellipsoid method is highly operator-dependent, since there were statistically different TKV estimates for both intra-and interobserver variability.

Seuss et al. [24] compared the kidney volumes obtained via a semi-automated segmentation method and via the ellipsoid method (using three different formulas) to reference volumes that were obtained using the manual segmentation method. There was no significant difference between volumes obtained via the semi-automated method and the reference volumes, and no significant intra- and inter-reader variability. The TKVs obtained via the ellipsoid method instead showed that there was a significant difference between the reference volumes and the inter-reader variability for this method. This difference was higher than for the reference method. Our results are in line with those of the study of Seuss et al. [24] concerning the significant difference between methods. However, the experience of Seuss et al. [24], in contrast with our results, showed that the kidney volumes obtained via the ellipsoid method were systematically too high compared to the reference volumes, but this report was not found in other studies in the literature.

Our study indeed has some limitations, since it is limited to a single center and two operators’ experience. Moreover, as it has been recently reported [30], there can be intrinsic TKV measurement biases related to the different MRI contrast mechanisms, and it should not be overlooked that some sequences are more susceptible than others to certain artifacts that could lead to greater imprecision in kidney volume measurement. In our experience, we performed the semi-automatic volumetric segmentation using on non-fat-saturated 3D coronal T1-weighted acquisitions, and the ellipsoid method using T2-weighted SSFSE and SSFP images. Any measurement biases due to the MRI contrast mechanism or pulse sequences could be a possible limitation of our study, which may be worth investigating in further studies.

## 5. Conclusions

This work had the objective of investigating performance in measuring the TKV of the semi-automatic volumetric segmentation method and the ellipsoid method.

The semi-automatic volumetric segmentation method has been confirmed to have excellent reliability and reproducibility compared to the ellipsoid method. Furthermore, the study showed, in accordance with data found in the literature, that the ellipsoid method is subject to more significant variability when used by operators with little experience. Another advantage of the semi-automatic volumetric segmentation method, unlike other segmentation methods described in the literature, is that it uses highly available software; said software is generally included in a standard dedicated radiological workstation.

The systematic use of the semi-automatic volumetric segmentation method in clinical practice, however, is limited by the longer time it requires. Several recent attempts to overcome the time consumption limit of segmentation methods using artificial intelligence tools can be found in the literature [31,32,33], and although further studies are needed, we believe that this is the right direction for future research in this field.

In our experience, however, the ellipsoid method applied to MR images has proved to be significantly faster, reasonably reliable, and accurate when performed by experienced operators.

However, manual or rather semi-automatic volumetric segmentation methods (like the one we tested) should, in our opinion, be used at least within the first evaluation of ADPKD patients, given their greater accuracy, for better prognostic classification and a more certain therapeutic indication.

## Figures and Tables

**Table 1:** Characteristics of the study population.

Characteristics of the Study Population	
Patients/kidneys (*n*)	40/78
Male *n* (%)	18 (45%)
Female *n* (%)	22 (55%)
Mean age (years)	48.8 ± 10.5
SKV right (mL) *	614 (457–1371)
SKV left (mL) *	818 (523–1371)

* Expressed as median and interquartile range (IQR) based on the expert operator’s measurements calculated using the semi-automatic volumetric segmentation method.

**Table 2:** MR protocol.

	TR (ms)	TE (ms)	Thickness (mm)	Gap (mm)	FOV	Matrix	NEX
Axial 2D SSFP	T2/T1	Steady state	4	0.4	36–40	224 × 320	1
Coronal 2D SSFP	T2/T1	Steady state	4	0.4	38–44	224 × 320	1
Axial LAVA	2–6	2–4	4	−2	40–48	320 × 192	-
Coronal T2 SSFSE	611	45–60 min	4	0.6	40–48	288 × 224	-
Sagittal T2 SSFSE	611	45–60 min	4	0.6	40–48	288 × 224	-
Coronal 3D Dual Echo	6–8 min	2.176 and 5.104	5.4	−2.7	36–44	320 × 192	-

**Table 3:** Mean percentage error of kidney volume computation using the ellipsoid method versus the semi-automatic volumetric segmentation method, calculated separately for the two operators and for the average of their measurements. Values are given as mean ± standard deviation.

	eSKV Mean Error, %	eTKV Mean Error, %
**Operator 1 (expert)**	−2.74 ± 11.79	−2.6 ± 8.74
**Operator 2 (beginner)**	3.25 ± 10.02	3.41 ± 8.29
**Average of operators**	0.27 ± 9.79	0.41 ± 7.76

**Table 4:** Comparison between methods.

	Comparison between Methods			
	Semi-Automatic Volumetric Segmentation vs. Ellipsoid Method			
	Operator 1		Operator 2	
	SKV	TKV	SKV	TKV
***p*** **-value**	0.0371	0.0416	0.0034	0.0171
**Median difference**	22.629	48.428	−29.515	−61.8575
**95% Confidence Interval**	2.04 to 43.809	1.5735 to 100.5375	−54.848 to −8.828	−123.327 to −9.862

**Table 5:** Comparison between operators.

	Comparison between Operators			
	Operator 1 vs. Operator 2			
	Semi-Automatic Volumetric Segmentation		Ellipsoid Method	
	SKV	TKV	SKV	TKV
***p*** **-value**	0.7742	0.948	<0.0001	<0.0001
**Median difference**	0.639	0.5125	−41.15	−89.45
**95% Confidence Interval**	−5.253 to 7.423	−14.027 to 16.8425	−62.25 to −25.4	−154.25 to −50.05

## Data Availability

Data supporting the findings of this study are available within the paper; some of them are not publicly available due to reasons of sensitivity and are available from the corresponding author upon reasonable request.

## Note

This research article is the prosecution and expansion of a previous work presented as scientific poster at European Congress of Radiology (ECR) 2020. The permission has been granted, since that copyright remains with the original authors (who are included in this extended version). Reference: C. Campisi, A. G. Torcitto, C. Marcantoni, S. Di Pietro, G. Failla, M. Veroux, L. Saporito, A. Basile, S. Palmucci. (2020) Calculation of kidney volumes with Magnetic Resonance in patients with autosomal dominant polycystic kidney disease: comparison between methods. ECR 2020 Poster C-15077. DOI: 10.26044/ecr2020/C-15077. © European Society of Radiology.
